# Testosterone, cortisol, hGH, and IGF‐1 levels in an Italian female elite volleyball team

**DOI:** 10.1002/hsr2.32

**Published:** 2018-03-09

**Authors:** Laura Roli, Sara De Vincentis, Marco Bruno Luigi Rocchi, Tommaso Trenti, Maria Cristina De Santis, Gustavo Savino

**Affiliations:** ^1^ Department of Laboratory Medicine and Pathology Azienda USL of Modena Italy; ^2^ Unit of Endocrinology, Department of Biomedical, Metabolic and Neural Sciences University of Modena and Reggio Emilia Italy; ^3^ Department of Medicine, Endocrinology, Metabolism and Geriatrics Azienda Ospedaliero‐Universitaria of Modena Italy; ^4^ Department of Biomolecular Science, Unit of Biostatistics University “Carlo Bo” of Urbino Italy; ^5^ Department of Public Healthcare, Sport Medicine Service Azienda USL of Modena Italy

**Keywords:** female athletes, reference intervals, testosterone/cortisol (T/C) ratio, overtraining

## Abstract

**Purpose:**

To assess the transferability of the reference intervals (RI) of testosterone (T), cortisol (C), human growth hormone (hGH), and insulin‐like growth factor (IGF)‐1, calculated on a normal healthy population, to a population of female elite volleyball players. Secondary aim of this study is the evaluation of the T/C ratio as predictive tool of overtraining during the annual regular season.

**Methods:**

A retrospective, longitudinal, observational study was performed, enrolling 58 professional female volleyball players periodically evaluated during the regular sportive season, which lasts from September to May.

**Results:**

Statistically significant differences between the volleyball players and reference populations for T (P = .010), C (P < .001), and IGF‐1 (P < .001) were found. Three different statistical approaches to calculate the RI in the athlete group showed a high degree of concordance and pointed out a shift upwards of both lower and upper reference limits. The T/C ratio significantly changed among visits (P = .009). In particular, an overall decrease of about 30% was observed for this ratio during the season, suggesting a state of overtraining.

**Conclusion:**

T, C, hGH, and IGF‐1 reference values calculated on elite volleyball female players are higher than those of the reference population used in normal clinical practice, suggesting that the health status of highly trained subjects needs the definition of tailored RI for these variables. Moreover, the utility of T/C ratio in the evaluation of overtraining is confirmed.

## Abstract 2

**Purpose**

To assess the transferability of the reference intervals (RI) of testosterone (T), cortisol (C), human growth hormone (hGH), and insulin‐like growth factor (IGF)‐1, calculated on a normal healthy population, to a population of female elite volleyball players. Secondary aim of this study is the evaluation of the T/C ratio as predictive tool of overtraining during the annual regular season.

**Methods**

A retrospective, longitudinal, observational study was performed, enrolling 58 professional female volleyball players periodically evaluated during the regular sportive season, which lasts from September to May.

**Results**

Statistically significant differences between the volleyball players and reference populations for T (P = .010), C (P < .001), and IGF‐1 (P < .001) were found. Three different statistical approaches to calculate the RI in the athlete group showed a high degree of concordance and pointed out a shift upwards of both lower and upper reference limits. The T/C ratio significantly changed among visits (P = .009). In particular, an overall decrease of about 30% was observed for this ratio during the season, suggesting a state of overtraining.

**Conclusion**

T, C, hGH, and IGF‐1 reference values calculated on elite volleyball female players are higher than those of the reference population used in normal clinical practice, suggesting that the health status of highly trained subjects needs the definition of tailored RI for these variables. Moreover, the utility of T/C ratio in the evaluation of overtraining is confirmed.

## 1 INTRODUCTION

The interest in hormonal changes in female athletes has rapidly increased in the last few years, which has led to an exponential rise in the number of studies available in the literature in this field. Sports medicine is focused on the athlete's health protection, through the use of reliable, predictive, and sensitive biochemical parameters, useful to detect overtraining, diseases, and doping. In recent years, increasingly more complex laboratory assays have been developed to detect the use of banned substances in sports medicine such as androgenic anabolic steroids, cortisol, and growth hormone (GH).^1^ Thus, the main objective of laboratory assays is to discriminate between a physiological variation of different biological variables, resulting from physical training, and the pathological change due to the illicit consumption of doping substances. Indeed, several changes in neuroendocrine axes occur in athletes, representing a physiologic adaptive response to a low energy state and to stressful physical and mental conditions.^2^ Adaptive responses could lead to alterations in tissue metabolism and may affect behavior, neurocognition, and mood.^3^, ^4^ However, the effects of training on hormonal secretion in the female athletes are yet to be completely elucidated because of the complexity of the hormonal secreting pattern.^5^, ^6^ Recently, the World Anti‐Doping Agency has developed a harmonized longitudinal profiling program based on continuous monitoring of the athletes over time, checking any significant change in blood and urinary biomarkers within their ranges.^1^

There is extensive literature regarding the peak concentrations of both human GH (hGH) and insulin‐like growth factor (IGF)‐1, which increase immediately after exercise.^7^, ^8^ Similarly, hGH levels correlate with training intensity, and the time to peak is shorter in women compared with men.^9^ Overall, training can lead to different effects, and the magnitude of the hGH release can be different according to the nature of the training itself (eg, for the same duration and total work effort, hGH levels are higher after a high‐intensity anaerobic work compared with low‐intensity anaerobic work),^10^ and to individual features such as age, sex, body composition, initial training, and fitness level.^10^ When elite athletes are compared with nonelite athletes and sedentary people, hGH levels are significantly higher, although no clear consensus is available concerning IGF‐1, which probably reflects the inter‐individual variability of this hormone.^11^, ^12^ Age‐dependent levels of hGH‐related markers are predictable in elite athletes, and they are independent of sporting category,^13^ suggesting that well‐trained people have their own serum and urinary ranges for both hGH and IGF‐1.^14^

Similarly, the role of cortisol (C) in the maintenance of body homeostasis in response to stressors, acute physical exercise, and chronic training is widely demonstrated both in athletes and sedentary people.^3^ Testosterone (T), like other anabolic‐androgenic steroids, enhances athletic performance in men and women through long‐term anabolic actions, as well as through rapid effects on behavior.^15^ Indeed, T production is dynamically regulated by both exercise and winning in competition. Bhasin et al^16^ described that T stimulates muscle mass and reduces body fat. C and T show a similar action supporting the neuromuscular system under stressful factors, promoting metabolic adaptive response and sustaining cognitive performances related to executive functions. More recent studies have demonstrated that androgens seem to act on specific substrates in the brain to increase aggression and motivation for competition.^17^

C and T play a significant role in protein and carbohydrate metabolism, working as competitive agonists at the receptor level of muscular cells.^18^ Thus, the T/C ratio seems to be a good indicator of the anabolic/catabolic balance, showing a significant decrease according to workout intensity and duration,^18^, ^19^, ^20^, ^21^ representing a useful tool in the early detection of overtraining syndrome.

On the basis of this evidence, we hypothesized that a well‐trained population of young female subjects might need a new definition of normal serum range levels for all of the aforementioned hormones. The availability of accurate personalized reference intervals could help clinicians assess the athlete's health status, avoiding any additional clinical investigation that would be requested when an abnormal laboratory result is obtained compared with the “standard” reference intervals (RIs). Thus, this study aimed to assess the transferability of the RIs of T, C, hGH, and IGF‐1 calculated on a normal healthy population and used in our laboratory, to a population of female elite volleyball players. Moreover, the secondary aim of this study was to evaluate the T/C ratio as a predictive tool for overtraining during the annual regular season.

## 2 MATERIALS AND METHODS

### 2.1 Study design

A retrospective, longitudinal, observational study was performed in the Clinical Pathology Laboratory of the Ospedale Civile Sant'Agostino Estense of Modena, Italy, together with the Sport Medicine Service of Modena, responsible for the health care of the local elite female volleyball team playing in the first‐class Italian Championship.

Fifty‐eight female professional volleyball players belonging to the same elite team were followed. Their health status was periodically evaluated during the regular sportive season. The standard health monitoring protocol consisted of 4 visits per season. Players were evaluated at the beginning of training (visit 1—September), at the beginning of the regular season (visit 2—November), in its middle (visit 3—January or February), and at the end (visit 4—May). During each clinical evaluation, a blood sample was taken at 8:00 to 9:00 am after an overnight fast, for hormonal laboratory tests.

To ensure statistical validity of the results by recruiting a large number of samples, laboratory data were collected over the course of 3 consecutive sportive seasons, from the middle season 2013 to the end of sportive season 2016, which corresponded to 9 routine clinical evaluations, for an overall number of samples of 132.

All players were consecutively enrolled in the study without applying any inclusion or exclusion criteria. They did not receive any drug that could interfere with their hormonal status nor were subjected to any controlled diet.

All subjects provided informed consent to the blood collection from the physician of the Sports Medicine Service for the control and monitoring of their health status. The Research and Innovation office, together with the institutional authority of laboratory, approved the use of data for the study.

### 2.2 Laboratory tests

Serum T, C, hGH, and IGF‐1 levels were measured with chemiluminescent immuno‐assays performed on completely automated platforms routinely used in our laboratory. T and C were analyzed by Architect 2nd Generation Testosterone (Abbott GmbH & Co, Germany) and Access Cortisol (Beckman Coulter Inc, USA), respectively. Similarly, hGH and IGF‐1 were analyzed on UniCel DXI800 platform (Beckman Coulter Inc, USA) and Liajson XL (Diasorin S.p.A., Saluggia, Italy), respectively.

The Laboratory adopted the manufacturers' suggested RIs based on female healthy populations; the information about these “reference populations” reported in Tables 1 and 2 were provided by Abbott GmbH (T and C), Beckman Coulter Inc (hGH), and Diasorin S.p.A (IGF). These assay‐calibrated RIs were as follows: T 0.35 to 2.08 nmol/L (Architect System. 2nd Generation Testosterone. REF 2P13, G6‐0507/R01. B2P1W4. Abbott), C 184.92 to 623.76 nmol/L (Access Cortisol, Istruzioni per l'uso A33262 J IT, 12/2016), and hGH 0.01 to 3.60 μg/L (Access Ultrasensitive hGH, Istruzioni per l'uso A38028 E IT, 12/2016). The IGF‐1 age‐related RI were the following: age range from 13 to 15 years, 104.00 to 591.00 μg/L; age from 15 to 17 years, 121.00 to 524.00 μg/L; age range from 17 to 20 years, 120.00 to 399.00 μg/L; age range from 20 to 30 years, 109.00 to 293.00 μg/L; age range from 30 to 40 years, 89.00 to 274.00 μg/L (Liaison IGF‐I. Ref. 313231. IT‐3‐2014‐04‐08).

**Table 1:** Demographics and testosterone (T), cortisol (C), human growth hormone (hGH) parameters of the 58 volleyball players enrolled in this study

	Female Volleyball Players Demographics and Hormonal Data				Female Reference Populations^d^ Demographics and Hormonal Data				
	Age Observed Range, y	Mean ± SD	Median Value (Observed Range)	Calculated RI	Age Observed Range, y	Mean ± SD	Median Value (Observed Range)	In use RI	*P* value
T, mmol/L C, mmol/L hGH, μg/L	24.15 ± 5.72 (15.18‐37.15)	1.22 ± 0.49 (n = 132) 502.32 ± 116.2 (n = 132) 2.85 ± 4.51 (n = 132)	1.04 (0.45‐3.51) 484.38 (264.96‐855.60) 0.68 (0.07‐24.25)	0.56‐2.39^a^ 0.52‐2.43^b^ 0.56‐2.47^c^ 311.33‐765.07^a^ 301.67‐778.32^b^ 309.12‐770.32^c^ 0.061‐28.58^a^ 0.076‐17.71^b^ 0.057‐31.08^c^	21‐49 NA 21‐73	0.94 ± 0.42 (n = 129) 359.63 ± 118.96 (n = NA) 0.57 ± 0.96 (n = 232)	NA (0.24‐2.74) NA (NA) NA (NA)	0.35‐2.08 184.92‐623.76 0.01‐3.6	.01 <.0001 <.0001

Reported *P* values were derived from the comparison between volleyball players and the female reference populations, performed using 1‐sample *t* test. Note that “n” indicates number of observations.

Abbreviations: C, cortisol; hGH, human growth hormone; NA, not available data; RI, reference interval; T, testosterone.

a RI calculated applying statistical method 1 (normal distribution).

b RI calculated applying statistical method 2 (quantile method).

c RI calculated applying statistical method 3 (robust statistical method).

d Reference population data were provided by Abbott GmbH (T and C) and Beckman Coulter Inc. (hGH). Architect System. 2nd Generation Testosterone. REF 2P13, G6‐0507/R01. B2P1W4, Abbott; Access Cortisol, Istruzioni per l'uso A33262 J IT, Abbott; 12/2016. Access Ultrasensitive hGH, Istruzioni per l'uso A38028 E IT, 12/2016, Beckman Coulter

**Table 2:** Demographics and IGF‐1 centiles calculated for the volleyball female team, and those used in the laboratory based on a healthy female population reference

IGF‐1	Female Volleyball Players	Female Reference Population^a^	*P* value
Number of observations (N)	132	1911	
Age observed range, y	15.18‐37.15	0‐57	
2.5th‐97.5th centiles, μg/L [20 y age]	191‐536	109‐372	<.0001
2.5th‐97.5th centiles, μg/L [25 y age]	146‐454	100‐311	<.0001
2.5th‐97.5th centiles, μg/L [30 y age]	136‐363	89‐290	<.0001
2.5th‐97.5th centiles, μg/L [35 y age]	154‐281	81‐278	<.0001

a Reference population data were provided by Diasorin S.p.A. Liaison IGF‐I. Ref. 313231. IT‐3‐2014‐04‐08.

Reported *P* values were derived from the comparison between volleyball players and the female reference populations using 1‐sample *t* test.

### 2.3 Statistical analysis

To determine the reference intervals, 3 different approaches were applied. (1) A normal distribution was assumed after Box‐Cox transformation of data (method 1); this method is based on the following transformation: *x*_transf_ *=* [(*x* + *c*)*λ−1*]/*λ*, when *λ* ≠ *0; x*_transf_ *= ln*(*x* + *c*), when *λ = 0*, where *x* is the original variable, *x*_*transf*_ is the transformed variable, *c* is a constant, and *λ* is the transformation parameter, estimated using the likelihood function.^22^

The assumption of normality, before and after transformation, was verified by D'Agostino‐Pearson test.^23^ On the basis of the assumption of normality, the reference interval (RI) could be calculated as follows: *RI = ± z*_*α/2*_ *σ*, *w*here μ is the mean, σ is the standard deviation, and *z*_*α/2*_ is the (*1*−*α/2*)th percentile of the Normal standard distribution.

(2) A quantile (or percentile) method was applied (method 2) according to NCCLS and Clinical and Laboratory Standards Institute (CLSI) guidelines C28‐A2 and C28‐A3.^24^ In this method, percentiles are calculated as the observations corresponding to the rank (ie, the position):

*r = p* (*n* + *1*), where *p* is the percentile expressed in the range [0,1], and *n* is the number of observations.^24^, ^25^, ^26^

(3) A robust statistical method was used (method 3), in which the confidence intervals for the RI are estimated using a bootstrapping procedure.^27^ This method is also recommended by CLSI Guidelines C28‐A3.^24^

The calculated percentile of T, C, and hGH was compared with the related laboratory's suggested values, and the ratio calculated/suggested, as indicated by Horowitz, was provided.^28^

Moreover, the percentage of reference measures outside the 97.5th centile of the laboratory's limits was calculated, according to Horowitz.^28^

For the analysis of IGF‐1, being this variable age‐related, polynomial functions were used, both for mean and for standard deviation, to estimate the reference values for different ages.^29^

The methodology performed to obtain a continuous age‐related reference interval is based on the following steps:

1. If the distribution of the considered variable shows skewness at different ages, the variable is transformed using a Box‐Cox transformation.
2. The transformed variable is fitted on age using a weighted polynomial regression model.
3. The residuals of this regression model are analyzed.
4. The absolute residuals, multiplied by are fitted on age using weighted polynomial regression. This second model provides the standard deviation of the transformed variable as a function of age *σ*_age_.
5. For each age value in the observed range, the reference interval RI is calculated according to this formula *RI*_age_ *=* μ_age_ *+ z*_*α/2*_ *σ*_age_. Obviously, if the variable has been initially transformed, it has to be back‐transformed to the original scale.

A comparison between volleyball players and the female reference populations was performed using 1‐sample *t* test. Because of the dependence of data collected from the same subjects at different times, all data were adjusted through a reduction, which provides new statistics that could be combined in a second‐order multilevel analysis.^30^ This reduction was performed weighting the original data according to the intraclass correlation coefficient, by the so‐called empty model, in which the level of the variable is obtained by adding the general mean, the casual effects at the group level, and the casual effects at the individual level.^31^

For the secondary aims, the distribution of hormonal variables was evaluated by Kolomogorov‐Smirnov test. Differences among visits were evaluated by univariate ANOVA for repeated measures, after verification of parametric assumptions, through Kolmogorov‐Smirnov test and Hartley's Fmax test; alternatively, if these assumptions were not met, the nonparametric Friedman test was used. Post‐hoc test was performed by Dunnett test.

Comparison between categorical variables was performed by chi‐square test. Relation between variables was evaluated using Pearson's coefficient correlation. Statistical analyses were performed using MedCalc Statistical Software version 15.8, SPSS (Statistical Package for the Social Sciences) software version 21.0, and a dedicated spreadsheet (Excel). For all comparisons, *P* values <.05 were considered statistically significant. The confidence interval (90% CI) width of the calculated limits of the RI was considered acceptable when it was less than 0.2 times the width of the RI.^24^

## 3 RESULTS

### 3.1 Hormonal levels at baseline

Three samples, belonging to 3 different players and corresponding to 2.27% of cases, had T values above the upper limit of the RI used in the laboratory, whereas 20 samples (13 players), 15.15% of cases, showed a C concentration above the RI used in the laboratory.

In 24.24% of cases, corresponding to 32 samples and 24 players, hGH hormone levels were above the higher limit of the RI. Thirty‐four samples (20 athletes), corresponding to 25.76% of the cases, showed IGF‐1 serum levels higher than the upper limit of the age‐dependent RI. In Table 1, the age ranges and the T, C, and hGH levels of the study population and of the reference are reported. In Table 2, the age ranges and the IGF‐1 centiles of the study population and reference population are reported.

### 3.2 Hormone reference ranges in the female volleyball players

T, C, and hGH RIs were calculated with a normal distribution‐based method, a nonparametric percentile method, and a robust method, as detailed in “Methods” (Table 1). Table 2 summarizes the age‐related IGF‐1 centiles. The study population was significantly different from the reference population regarding T (*P* = .01), C (*P* < .0001), and hGH (*P* < .0001) (Table 1). Each calculated IGF‐1 centile was significantly different from the corresponding centile of the reference population; the *P* value was <.0001 for all the age ranges taken into consideration (Table 2). These findings suggest the group of athletes investigated in this study have T, C, hGH, and IGF‐1 serum levels different from the “normal” female populations on which the RIs in use in our laboratory are defined.

#### 3.2.1 Testosterone and cortisol

**Figure 1:**
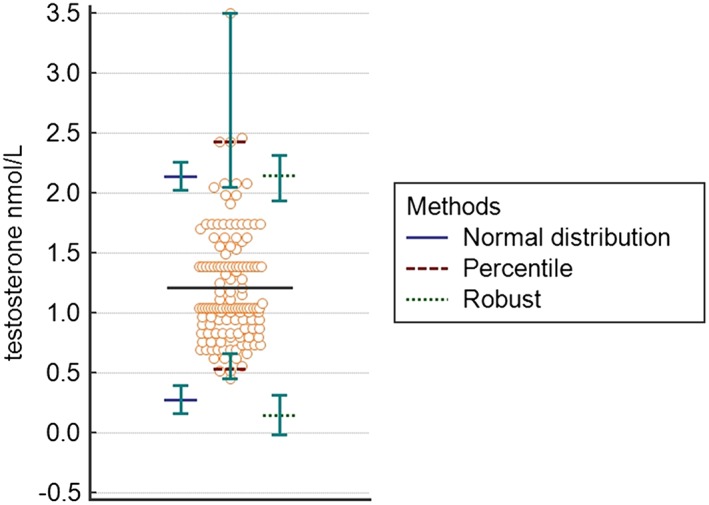
Dot plot for testosterone and reference intervals, according to the 3 different statistical methods used in the study (normal distribution, percentile, and robust). Each circle dot represents an athlete's hormonal data. Horizontal black line indicates the mean value of testosterone serum level in the study population. Upper and lower limits of the reference intervals calculated with 3 different statistical methods (see box in the figure) are reported with their corresponding 90% CI

**Figure 2:**
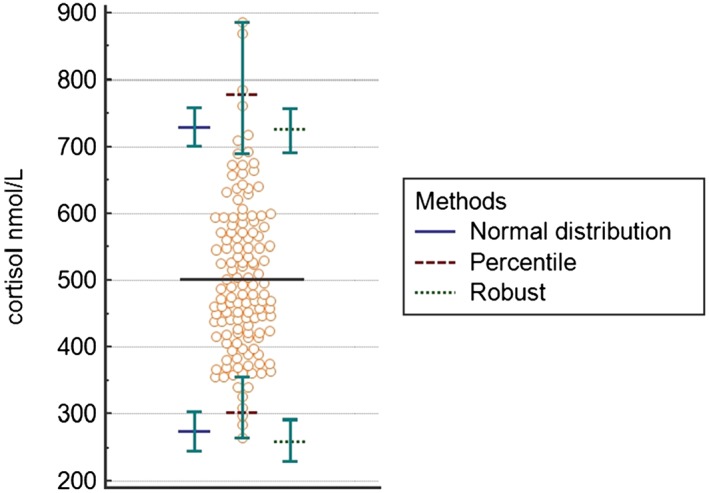
Dot plot for cortisol and reference intervals, according to the 3 different statistical methods used in the study (normal distribution, percentile, and robust). Each circle dot represents an athlete's hormonal data. Horizontal black line indicates the mean value of cortisol serum level in the study population. Upper and lower limits of the reference intervals calculated with 3 different statistical methods are reported with their corresponding 90% CI

The 3 different statistical approaches showed a high degree of concordance of T reference range calculated on the athletes' data (Figure 1 and Table 1). The lower limits of T calculated with normal, percentile, and robust methods were 61%, 50%, and 60% higher, respectively, than the lower limit of the RI in use in the laboratory; the upper limits of T calculated with normal, percentile, and robust methods were 15%, 17%, and 19% higher, respectively, than the upper limit of the RI in use in the laboratory. Similarly, the 3 statistical approaches showed a high degree of concordance of the C reference ranges calculated on the athletes' data (Figure 2 and Table 1). The lower limits of C calculated with normal, percentile, and robust methods were 68%, 63%, and 67% higher, respectively, than the lower limit of the RI in use in the laboratory; the upper limits of C calculated with normal, percentile, and robust methods were 23%, 25%, and 24% higher, respectively, than the upper limit of the RI in use in the laboratory. Regardless of the statistical method used, 90% CI of the calculated lower limits for both hormones fulfilled the acceptability criteria. On the other hand, 90% CI of the calculated T upper limits did not meet criteria of acceptability. Regarding C, 90% CI of the upper limit was unacceptably wide only when calculated with the percentile method.

#### 3.2.2 hGH and IGF‐1

**Figure 3:**
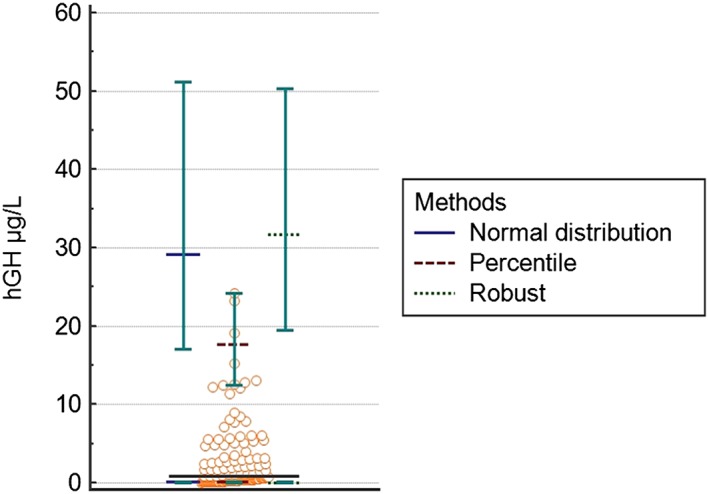
Dot plot for human growth hormone (hGH) and reference intervals, according to the 3 different statistical methods used in the study (normal distribution, percentile, and robust). Each circle dot represents an athlete's hormonal data. Horizontal black line indicates the mean value of hGH serum level in the study population. Upper and lower limits of the reference intervals calculated with 3 different statistical methods (see box in the figure) are reported with their corresponding 90%CI

hGH data were not normally distributed, and the 3 statistical approaches produced RIs significantly higher than those in use by the laboratory (*P* < .001) (Figure 3 and Table 1).

**Figure 4:**
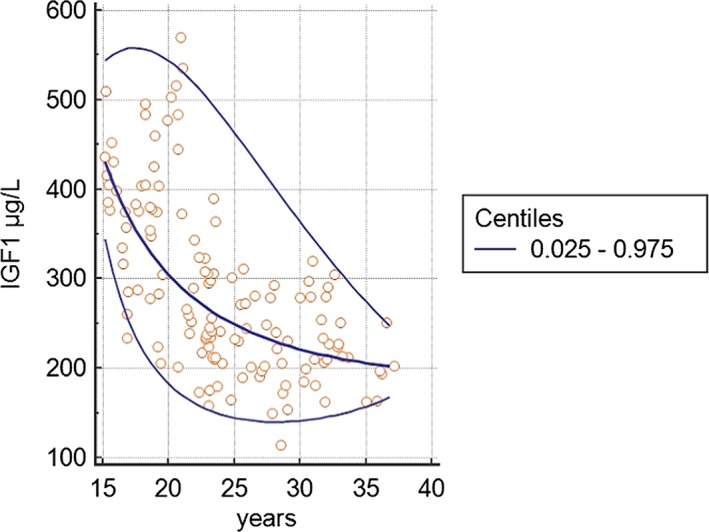
Age‐dependent insulin growth factor‐1 (IGF‐1) levels. Scatter diagram of the measurements versus age with the calculated mean (central line) and centile curves. Each circle represents an athlete's hormonal data

The 3 statistical approaches produced concordant lower limits of hGH: they were, on average, 100% higher than the lower limit of the RI in use in the laboratory; the upper limits of hGH calculated with normal, percentile, and robust methods were 164%, 64%, and 187% higher, respectively, than the upper limit of the RI in use in the laboratory. It must be pointed out that the standard deviation was almost twice the average of the hormone concentration, demonstrating a large intra‐ and inter‐individual variability that could justify this finding. 90% CI of the lowest value fitted the acceptability criterion, regardless of the statistical method used, while 90% CI of the upper limit was larger than 0.2 times the RI width. Figure 4 shows the relation between athletes' age and IGF‐1 serum concentration. Table 2 reports the IGF‐1 age‐related RI calculated using the volleyball players' data, and they are compared with the matching‐aged RI of the reference population. Significant differences were found for each age range evaluated (*P* < .001).

#### 3.2.3 Hormonal trend during regular season

T and C serum levels significantly changed (*P* = .013 and *P* = .009, respectively) among visits, whereas GH and IGF‐1 did not. At post‐hoc test, T serum levels were significantly higher at visit 4 than at visits 1 and 3 (*P* = .024 and *P* = .016, respectively); levels in visit 1 were significantly lower than at visit 2 (*P* = .029); and levels in visit 2 were significantly higher than at visit 3 (*P* = .017), altogether suggesting that T serum levels are higher at the beginning and at the end of the regular season. Regarding C, at post‐hoc test, its levels were significantly lower at visit 1 than at visit 2 (*P* = .003), and they were significantly higher at visit 2 than at visits 3 and 4 (*P* = .049 and *P* = .005, respectively), suggesting that cortisol is higher when the regular season begins and its levels progressively decrease thereafter.

**Figure 5:**
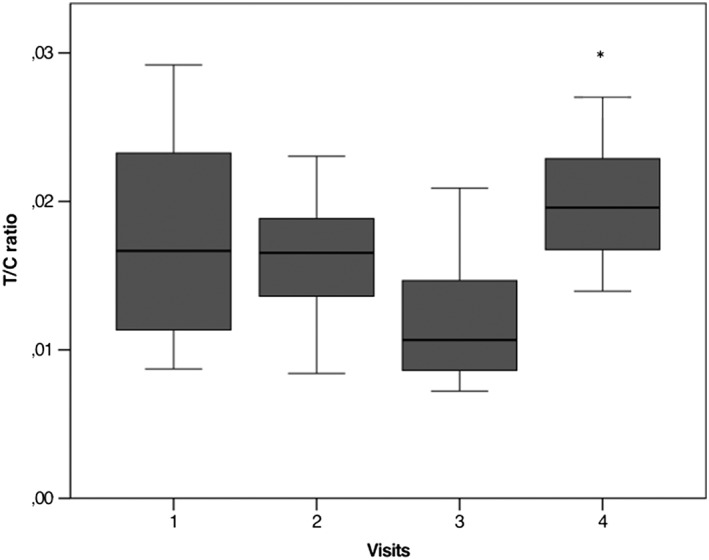
Testosterone (T)/cortisol (C) ratio among visits. The dark line in the middle of the boxes represents the median of T/C ratio; the bottom of the box indicates the 25th percentile, while the top of the box represents the 75th percentile; whiskers extend to the minimum and maximum values. (*) At the nonparametric Friedman test followed by Dunnett post‐hoc test, T to C ratio was higher at visit 4 than at visit 3 (*P* = .003)

The T/C ratio has been used as a performance index for athletes.^18^ The T/C ratio significantly changed among visits (*P* = .009) (Figure 5). At post‐hoc test, it showed higher values at visit 4 than at visit 3 (*P* = .003) (Figure 5). However, the T/C ratio decreased from visit 1 to visit 3, although not in a statistically significant manner. Several authors have proposed that a T/C decrease of more than 30% suggests a state of overtraining.^18^, ^19^, ^20^ In our study, T/C ratio decreased from 0.017 at visit 1 to 0.012 at visit 3, corresponding to a decrease of about 30% (Figure 5).

Considering GH, we only had GH serum levels from 14 subjects. We found a mean GH value higher than the upper limit of the reference range of our laboratory (3.6 μg/L); accordingly, IGF‐1 serum levels were, similarly, at the upper limit of reference range. However, neither GH nor IGF‐1 significantly changed among visits. We found that 5 of 14 athletes had GH levels above the normal range at visit 1 (35.7%). We subdivided subjects into 2 groups according to (1) GH < 3.6 μg/L (group A) and (2) GH > 3.6 μg/L (group B). After removal of subjects with increased basal hGH serum levels, both hGH and IGF‐1 did not change during the regular season.

Considering all hormones evaluated, no correlations were found at baseline. At visit 2, representing the start of the regular season, cortisol was directly related to testosterone (*R* = .572, *P* = .039) and GH (*R* = .560, *P* = .037). Moreover, testosterone was directly related to cortisol and GH (*R* = .694, *P* = .010). At visit 3, all of these relationships were lost and a direct correlation between GH and IGF‐1 was observed (*R* = .449, *P* = .019). Finally, at the end of the regular season (visit 4), GH was directly related to testosterone (*R* = .836, *P* = .038).

## 4 DISCUSSION

In this study, we demonstrate that serum T, C, hGH, and IGF‐1 reference ranges calculated using the data of an elite volleyball female team are higher than those we routinely use in clinical practice, which are derived from a “reference” of healthy female individuals. This result, consistent across different statistical methods, suggests that the young female athlete is constitutively different from the normal female population regarding the levels of these hormones.

In general, the 4 hormone levels are higher in a group of 58 young female volleyball players with ages ranging from 15.18 to 37.15 years, compared with healthy subjects. This finding suggests that physical exercise induces long‐term hormonal changes in highly trained athletes, and as consequence, there is a need for laboratory reference ranges to be tailored to this specific population. This evidence should be taken into consideration by laboratories that work with sports medicine services. Indeed, studies based on a large number of athletes practicing different sports highlighted the need to establish normative serum androgen levels in elite female athletes, with the precise purpose of helping in the development of a blood steroidal module in the field of Athlete Biological Passport.^32^

These new concepts are also needed to refine more evidence‐based recommendations concerning hyperandrogenism in female athletes.^32^ Our study is the first one properly designed to define appropriate laboratory reference values for the clinical assessment of the female athlete heath status.

Notwithstanding the consistency of the results obtained with the 3 statistical methods used to calculate the hGH RI, the robust method proposed by CLSI has provided the larger reference interval (0.057‐31.08 ng/mL), compared with those obtained through the normal distribution method (0.061‐28.58 ng/mL) and the quantile method (0.076‐17.71 ng/mL). This discrepancy could be explained by the use of a second‐order statistic. Moreover, we observed that the hGH data, both the raw data and after Box‐Cox transformation (ie, the most powerful technique of data transformation),^33^ were not normally distributed. Although CLSI proposes the so‐called robust method as standard procedure for nonnormality variables, our findings suggest that the most suitable method for the calculation of reference values appears to be the classical Efron's quantile method, under both normality and nonnormality of the raw data.

Notwithstanding that the RI upper limits of T, C, and hGH found in this study consistently describe a shift upwards of the RI, the large 90% CI, in particular as far as the hormone hGH concerns, suggests the need of a larger sampling of female volleyball players to obtain improved precision in the estimated upper limit of the RI. On the contrary, in this study we demonstrated the volleyball players population to have T, C, and hGH lower reference limits higher than those of “normal” female populations. The need of partitioning the IGF‐1 RI by age requires a larger number of subjects in each age range, so as to fulfill the recommendations of the CLSI standard. Moreover, we recently demonstrated that immunoenzymatic methods constitutively overestimate T detection, compared with mass spectrometry, although in a different clinical setting.^34^ Thus, this result should be better evaluated using different assays. Moreover, many variables, both related to female physiology and to lifestyle, can influence T circulating levels in female subjects and can underlie the difference we found. Enea C et al^6^ reviewed the biological factors affecting androgen levels in women, highlighting the complexity of the hormonal pattern in this sex. Unfortunately, we did not possess some useful information that could have helped explain some of the differences found, which is a limitation of our study. For instance, total T serum levels are higher in Caucasian women than in African and Hispanic women.^6^ The volleyball team we studied is entirely composed of Caucasian players, while the reference population is likely to have included women of different races. T declines with age^6^ and all of the elite players are within a narrow age range, while the reference population incorporates women from 21 years to 50 and older. There is evidence that T is directly influenced by alcohol assumption and diet; a high energy intake is directly related to T levels, or indirectly, through variation in SHBG.^6^ Moreover, androgens are menstrual cycle dependent and are influenced by contraceptives. All of these aspects should be considered in properly designed studies needed to better understand the reference values of T serum levels in this cohort of subjects.

The hGH RIs are significantly higher than those suggested for the general population. The 3 different statistical approaches provided highly consistent results, although the nonparametric percentile method provided a lower upper limit when compared with the other 2 statistics. We found a wide inter‐individual variability of the hormone, as the value of the SD exceeded the average serum concentration. We calculated IGF‐1 reference values in an age‐dependent manner and found that they are significantly higher than those in use in the laboratory.

We do not know the statistical method used by the manufacturers to establish the RIs of the normal population, but as we demonstrate in this study, different statistical methods provide consistent results. Accordingly, we can be confident that different statistical methods most likely do not account for the differences of hormonal RIs between reference and study population.

It is well known that the continuous, competitive, regular sport practice influences endocrine homeostasis through specific variation of total serum T and C levels. Here, we detect a C and T increase at the beginning of the season, representing the high intensity of physical exercise needed to start the regular season. Then, a slight decrease in C serum levels is observed during the year, while T fluctuates with a decrease in the middle of the season and an increase at the end. Indeed, muscular activity induces specific changes in endocrine function, to maintain body homeostasis.^35^ Acute activity leads to a C level increase, while regular continuative exercise modulates the elevation of C levels over time. Thus, the intensity of physical activity is able to influence the manner of C response. In our setting, training phase could be considered as acute exercise, with a significant increase of C levels. The final effect of training is the adaptation of endocrine functions to further muscular exercises, confirmed by the C decrease after training. This effect remains also when subdividing patients according to the role in the team, hypothesizing that the training activity is personalized to the role of the volleyball player. On the contrary, the interpretation of T level changes during physical activity, both in men and women, remains challenging.

The T/C ratio is a diagnostic tool proposed to evaluate overtraining in exercise in men.^20^ It is well known that C has a catabolic effect, whereas T is responsible for the stimulation of the anabolic process of skeletal muscle growth.^16^, ^18^ Their ratio is extremely important to evaluate endocrine homeostasis during acute and chronic exercise. Indeed, the T/C ratio represents an index of athletic performance and it decreases in our cohort of women in about 30% from the beginning of training to the middle of the regular season.^20^ This decrease suggests that athletes undergo overreached training during the regular season. On the contrary, the T/C ratio increases at the end of the season, returning to physiological levels. This increase suggests an adaptation at the end of the regular sportive year. The use of T/C ratio has recently been proposed also in female athletes,^36^ and here we confirm the possible use of the tool in this setting.

The hGH/IGF‐1 axis is affected during both the training phase and the regular season. This is confirmed after the exclusion of patients with elevated GH serum levels at baseline. Previously, Mejri et al^37^ found in 13 football players that GH levels were substantially higher at the beginning of the training phase and progressively decreased during the football season. However, in this previous study, blood samples were taken immediately after physical activity. In our study, we could confirm higher GH levels at the beginning of the training phase, although no differences were observed during the regular season. This result seems to be in accordance with previous findings in which the GH response is attenuated by a prior endurance exercise.^7^ Exercise induces both short effects on metabolism and long‐term effects on body composition and cardiac activity.^7^ Thus, we could speculate, first, that the training phase in our volleyball players does not represent an endurance activity, considering the stability of GH during the regular season. Second, the lack of GH changes could be related to the time interval between exercise and blood samples. A possible explanation for this trend of GH serum levels could be hypothesized, considering that GH levels start to increase 10 to 20 minutes after the onset of exercise and remained elevated only for 2 hours after the activity.^38^ In our study, blood samples were taken in the morning, after an overnight fast, but at least 48 hours after the physical exercise.

Our study has additional limitations. First, we evaluated hormone pattern only in 4 visits during the regular season. Second, we evaluate steroids using immunoenzymatic assays. It is well known that steroids, and especially T, are difficult to evaluate, and the gold‐standard method remains liquid chromatography‐mass spectrometry.^39^, ^40^

The second‐order multilevel analysis,^30^ which was applied to adjust the repeated measures in the volleyball players' population, assures that the average values are not affected by those subjects showing enhanced hormonal basal concentrations along the different phases of the sportive season. At the same time, this provides a sample data, which are representative of all the different, individual, adaptative changes occurring in response to both physical stressors (mainly level of health and training) and emotional stressors (mainly competitions) throughout the season.^18^, ^19^

Notwithstanding the need to confirm our results on a larger sample, in conclusion, we found that T, C, hGH, and IGF‐1 reference values calculated on elite volleyball female players are higher than those in use in the laboratory, suggesting the health status of these highly trained subjects need to be assessed using different RIs than those used in the general population. Moreover, we confirm the utility of T/C ration in the evaluation of overtraining.

### 4.1 Perspectives

The current retrospective, longitudinal, observational study on hormonal changes in a female elite volleyball team showed that well‐trained players have higher basal levels of T, C, hGH, and IGF‐1 than normal healthy female subjects. This suggests that continuous physical training leads to constitutive hormonal changes in female athletes, albeit with a large inter‐individual variability. Laboratory reference ranges tailored to this specific population should be made available to the sports medicine physician to ensure a correct clinical assessment of the athlete's health status. Indeed, referring to the RI routinely used in clinical practice can be misleading, given the implication that a high level of one of these hormones can have on further clinical investigations (and consequently on costs) and on the suspicion of illicit substances' consumption. Moreover, as RIs are assay dependent, they cannot be transferred from one laboratory to another unless the same assays are used; consensus RIs could be defined only by mass spectrometry. Finally, further studies including a larger population of female elite volleyball players are needed to confirm the RIs we calculated.

## CONFLICT OF INTEREST

The authors declare that no conflicts of interest are present. The results of the study are presented clearly, honestly, and without fabrication, falsification, or inappropriate data manipulation.

## FUNDING

None.

## AUTHOR CONTRIBUTIONS

Conceptualization: Laura Roli, Sara De Vincentis

Data curation: Laura Roli, Gustavo Savino

Formal analysis: Marco Bruno Luigi Rocchi, Sara De Vincentis

Writing – original draft preparation: Laura Roli, Sara De Vincentis

Writing – review and editing: Laura Roli, Sara De Vincentis, Tommaso Trenti, Maria Cristina De Santis, Gustavo Savino
